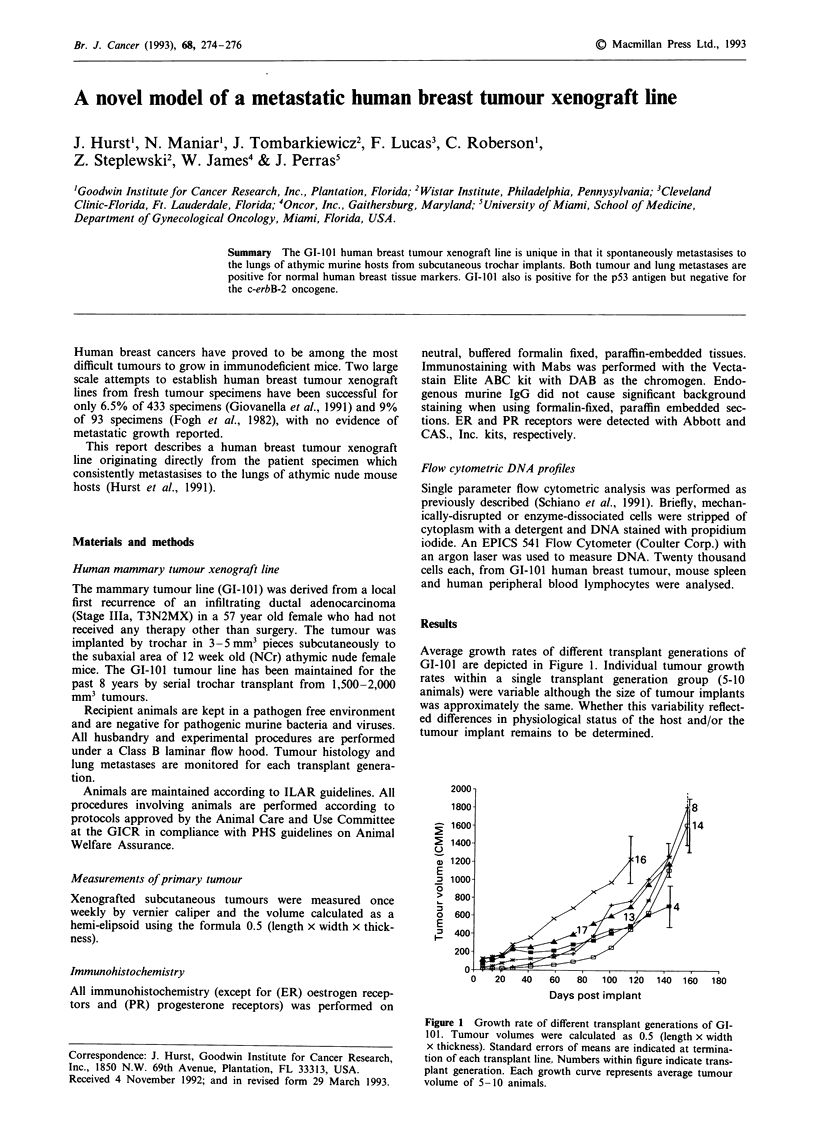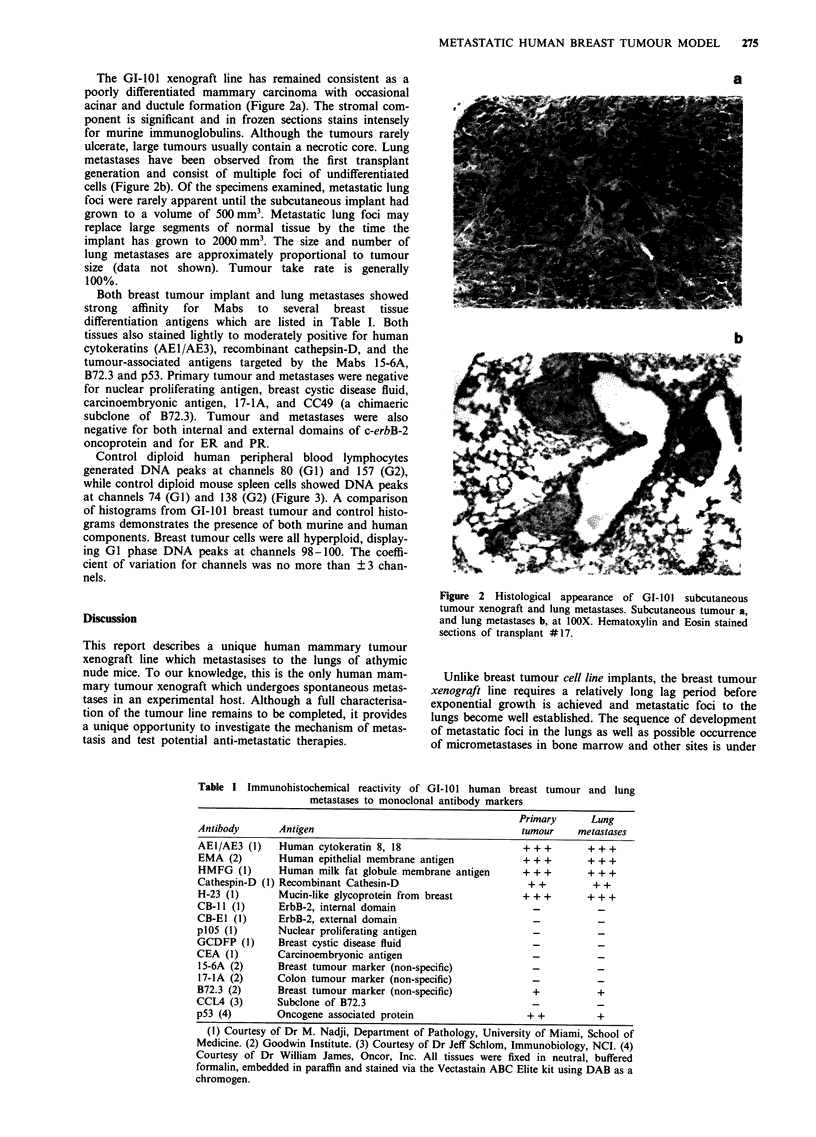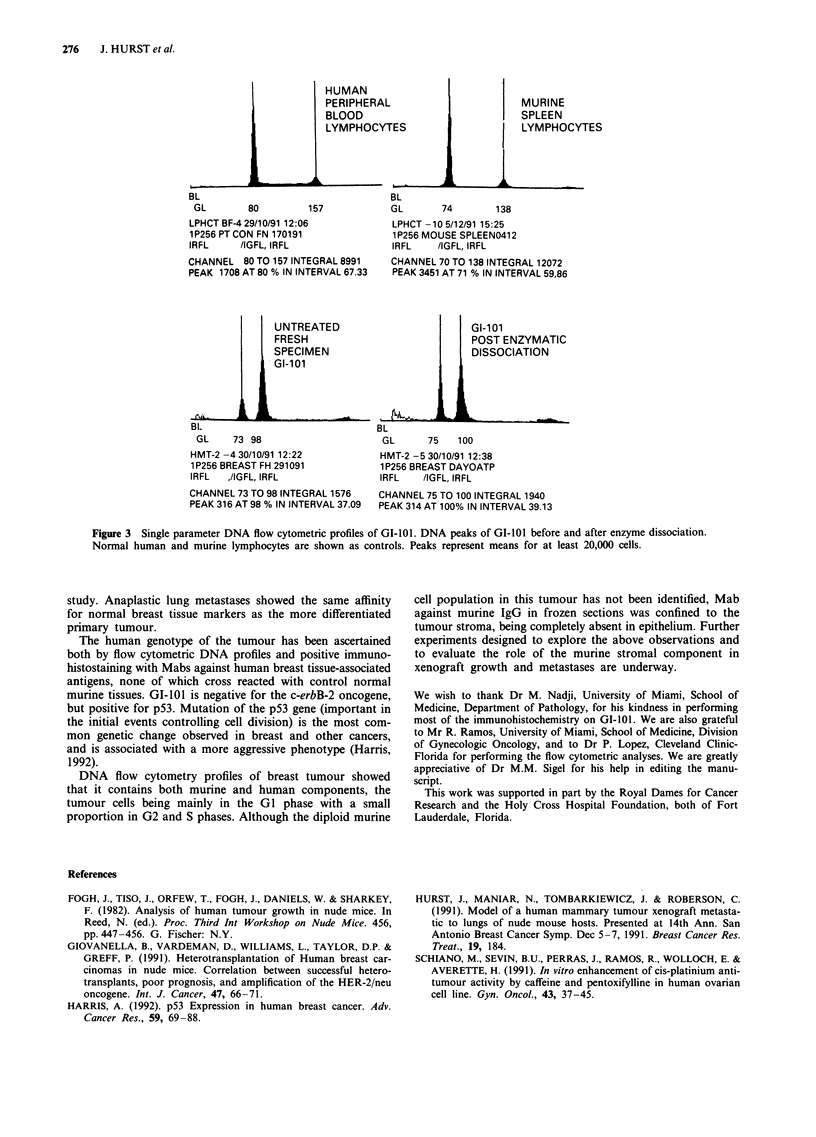# A novel model of a metastatic human breast tumour xenograft line.

**DOI:** 10.1038/bjc.1993.327

**Published:** 1993-08

**Authors:** J. Hurst, N. Maniar, J. Tombarkiewicz, F. Lucas, C. Roberson, Z. Steplewski, W. James, J. Perras

**Affiliations:** Goodwin Institute for Cancer Research, Inc., Plantation, Florida 33313.

## Abstract

**Images:**


*Br. J. Cancer* (1993), **68**, 274–276 © Macmillan Press Ltd., 1993

### A novel model of a metastatic human breast tumour xenograft line

J. Hurst[1], N. Maniar[1], J. Tombarkiewicz[2], F. Lucas[3], C. Roberson[1], Z. Steplewski[2], W. James[4] & J. Perras[5]

[1]*Goodwin Institute for Cancer Research, Inc., Plantation, Florida;* [2]*Wistar Institute, Philadelphia, Pennysylvania;* [3]*Cleveland Clinic-Florida, Ft. Lauderdale, Florida;* [4]*Oncor, Inc., Gaithersburg, Maryland;* [5]*University of Miami, School of Medicine, Department of Gynecological Oncology, Miami, Florida, USA.*

**Summary** The GI-101 human breast tumour xenograft line is unique in that it spontaneously metastasises to the lungs of athymic murine hosts from subcutaneous trochar implants. Both tumour and lung metastases are positive for normal human breast tissue markers. GI-101 also is positive for the p53 antigen but negative for the c-*erb*B-2 oncogene.

Human breast cancers have proved to be among the most difficult tumours to grow in immunodeficient mice. Two large scale attempts to establish human breast tumour xenograft lines from fresh tumour specimens have been successful for only 6.5% of 433 specimens (Giovanella *et al.*, 1991) and 9% of 93 specimens (Fogh *et al.*, 1982), with no evidence of metastatic growth reported.

This report describes a human breast tumour xenograft line originating directly from the patient specimen which consistently metastasises to the lungs of athymic nude mouse hosts (Hurst *et al.*, 1991).

### Materials and methods

### *Human mammary tumour xenograft line*

The mammary tumour line (GI-101) was derived from a local first recurrence of an infiltrating ductal adenocarcinoma (Stage IIIa, T3N2MX) in a 57 year old female who had not received any therapy other than surgery. The tumour was implanted by trochar in 3–5 $mm^3$ pieces subcutaneously to the subaxial area of 12 week old (NCr) athymic nude female mice. The GI-101 tumour line has been maintained for the past 8 years by serial trochar transplant from 1,500–2,000 $mm^3$ tumours.

Recipient animals are kept in a pathogen free environment and are negative for pathogenic murine bacteria and viruses. All husbandry and experimental procedures are performed under a Class B laminar flow hood. Tumour histology and lung metastases are monitored for each transplant generation.

Animals are maintained according to ILAR guidelines. All procedures involving animals are performed according to protocols approved by the Animal Care and Use Committee at the GICR in compliance with PHS guidelines on Animal Welfare Assurance.

### *Measurements of primary tumour*

Xenografted subcutaneous tumours were measured once weekly by vernier caliper and the volume calculated as a hemi-elipsoid using the formula 0.5 (length × width × thickness).

### *Immunohistochemistry*

All immunohistochemistry (except for (ER) oestrogen receptors and (PR) progesterone receptors) was performed on neutral, buffered formalin fixed, paraffin-embedded tissues. Immunostaining with Mabs was performed with the Vectastain Elite ABC kit with DAB as the chromogen. Endogenous murine IgG did not cause significant background staining when using formalin-fixed, paraffin embedded sections. ER and PR receptors were detected with Abbott and CAS., Inc. kits, respectively.

### *Flow cytometric DNA profiles*

Single parameter flow cytometric analysis was performed as previously described (Schiano *et al.*, 1991). Briefly, mechanically-disrupted or enzyme-dissociated cells were stripped of cytoplasm with a detergent and DNA stained with propidium iodide. An EPICS 541 Flow Cytometer (Coulter Corp.) with an argon laser was used to measure DNA. Twenty thousand cells each, from GI-101 human breast tumour, mouse spleen and human peripheral blood lymphocytes were analysed.

### Results

Average growth rates of different transplant generations of GI-101 are depicted in Figure 1. Individual tumour growth rates within a single transplant generation group (5-10 animals) were variable although the size of tumour implants was approximately the same. Whether this variability reflected differences in physiological status of the host and/or the tumour implant remains to be determined.

**Figure 1** Growth rate of different transplant generations of GI-101. Tumour volumes were calculated as 0.5 (length × width × thickness). Standard errors of means are indicated at termination of each transplant line. Numbers within figure indicate transplant generation. Each growth curve represents average tumour volume of 5–10 animals.

Correspondence: J. Hurst, Goodwin Institute for Cancer Research, Inc., 1850 N.W. 69th Avenue, Plantation, FL 33313, USA.
Received 4 November 1992; and in revised form 29 March 1993.

The GI-101 xenograft line has remained consistent as a poorly differentiated mammary carcinoma with occasional acinar and ductule formation (Figure 2a). The stromal component is significant and in frozen sections stains intensely for murine immunoglobulins. Although the tumours rarely ulcerate, large tumours usually contain a necrotic core. Lung metastases have been observed from the first transplant generation and consist of multiple foci of undifferentiated cells (Figure 2b). Of the specimens examined, metastatic lung foci were rarely apparent until the subcutaneous implant had grown to a volume of 500 $mm^3$. Metastatic lung foci may replace large segments of normal tissue by the time the implant has grown to 2000 $mm^3$. The size and number of lung metastases are approximately proportional to tumour size (data not shown). Tumour take rate is generally 100%.

Both breast tumour implant and lung metastases showed strong affinity for Mabs to several breast tissue differentiation antigens which are listed in Table I. Both tissues also stained lightly to moderately positive for human cytokeratins (AE1/AE3), recombinant cathepsin-D, and the tumour-associated antigens targeted by the Mabs 15-6A, B72.3 and p53. Primary tumour and metastases were negative for nuclear proliferating antigen, breast cystic disease fluid, carcinoembryonic antigen, 17-1A, and CC49 (a chimaeric subclone of B72.3). Tumour and metastases were also negative for both internal and external domains of c-*erb*B-2 oncoprotein and for ER and PR.

Control diploid human peripheral blood lymphocytes generated DNA peaks at channels 80 (G1) and 157 (G2), while control diploid mouse spleen cells showed DNA peaks at channels 74 (G1) and 138 (G2) (Figure 3). A comparison of histograms from GI-101 breast tumour and control histograms demonstrates the presence of both murine and human components. Breast tumour cells were all hyperploid, displaying G1 phase DNA peaks at channels 98–100. The coefficient of variation for channels was no more than ±3 channels.

**Figure 2** Histological appearance of GI-101 subcutaneous tumour xenograft and lung metastases. Subcutaneous tumour **a**, and lung metastases **b**, at 100X. Hematoxylin and Eosin stained sections of transplant #17.

### Discussion

This report describes a unique human mammary tumour xenograft line which metastasises to the lungs of athymic nude mice. To our knowledge, this is the only human mammary tumour xenograft which undergoes spontaneous metastases in an experimental host. Although a full characterisation of the tumour line remains to be completed, it provides a unique opportunity to investigate the mechanism of metastasis and test potential anti-metastatic therapies.

Unlike breast tumour *cell line* implants, the breast tumour *xenograft* line requires a relatively long lag period before exponential growth is achieved and metastatic foci to the lungs become well established. The sequence of development of metastatic foci in the lungs as well as possible occurrence of micrometastases in bone marrow and other sites is under

**Table I** Immunohistochemical reactivity of GI-101 human breast tumour and lung metastases to monoclonal antibody markers

| *Antibody* | *Antigen* | *Primary tumour* | *Lung metastases* |
|---|---|---|---|
| AE1/AE3 (1) | Human cytokeratin 8, 18 | +++ | +++ |
| EMA (2) | Human epithelial membrane antigen | +++ | +++ |
| HMFG (1) | Human milk fat globule membrane antigen | +++ | +++ |
| Cathespin-D (1) | Recombinant Cathesin-D | ++ | ++ |
| H-23 (1) | Mucin-like glycoprotein from breast | +++ | +++ |
| CB-11 (1) | ErbB-2, internal domain | – | – |
| CB-E1 (1) | ErbB-2, external domain | – | – |
| p105 (1) | Nuclear proliferating antigen | – | – |
| GCDFP (1) | Breast cystic disease fluid | – | – |
| CEA (1) | Carcinoembryonic antigen | – | – |
| 15-6A (2) | Breast tumour marker (non-specific) | – | – |
| 17-1A (2) | Colon tumour marker (non-specific) | – | – |
| B72.3 (2) | Breast tumour marker (non-specific) | + | + |
| CCL4 (3) | Subclone of B72.3 | – | – |
| p53 (4) | Oncogene associated protein | ++ | + |

(1) Courtesy of Dr M. Nadji, Department of Pathology, University of Miami, School of Medicine. (2) Goodwin Institute. (3) Courtesy of Dr Jeff Schlom, Immunobiology, NCI. (4) Courtesy of Dr William James, Oncor, Inc. All tissues were fixed in neutral, buffered formalin, embedded in paraffin and stained via the Vectastain ABC Elite kit using DAB as a chromogen.

**Figure 3** Single parameter DNA flow cytometric profiles of GI-101. DNA peaks of GI-101 before and after enzyme dissociation. Normal human and murine lymphocytes are shown as controls. Peaks represent means for at least 20,000 cells.

study. Anaplastic lung metastases showed the same affinity for normal breast tissue markers as the more differentiated primary tumour.

The human genotype of the tumour has been ascertained both by flow cytometric DNA profiles and positive immunohistostaining with Mabs against human breast tissue-associated antigens, none of which cross reacted with control normal murine tissues. GI-101 is negative for the c-*erb*B-2 oncogene, but positive for p53. Mutation of the p53 gene (important in the initial events controlling cell division) is the most common genetic change observed in breast and other cancers, and is associated with a more aggressive phenotype (Harris, 1992).

DNA flow cytometry profiles of breast tumour showed that it contains both murine and human components, the tumour cells being mainly in the G1 phase with a small proportion in G2 and S phases. Although the diploid murine cell population in this tumour has not been identified, Mab against murine IgG in frozen sections was confined to the tumour stroma, being completely absent in epithelium. Further experiments designed to explore the above observations and to evaluate the role of the murine stromal component in xenograft growth and metastases are underway.

We wish to thank Dr M. Nadji, University of Miami, School of Medicine, Department of Pathology, for his kindness in performing most of the immunohistochemistry on GI-101. We are also grateful to Mr R. Ramos, University of Miami, School of Medicine, Division of Gynecologic Oncology, and to Dr P. Lopez, Cleveland Clinic-Florida for performing the flow cytometric analyses. We are greatly appreciative of Dr M.M. Sigel for his help in editing the manuscript.

This work was supported in part by the Royal Dames for Cancer Research and the Holy Cross Hospital Foundation, both of Fort Lauderdale, Florida.

### References

FOGH, J., TISO, J., ORFEW, T., FOGH, J., DANIELS, W. & SHARKEY, F. (1982). Analysis of human tumour growth in nude mice. In Reed, N. (ed.). *Proc. Third Int Workshop on Nude Mice*. 456, pp. 447–456. G. Fischer: N.Y.

GIOVANELLA, B., VARDEMAN, D., WILLIAMS, L., TAYLOR, D.P. & GREFF, P. (1991). Heterotransplantation of Human breast carcinomas in nude mice. Correlation between successful heterotransplants, poor prognosis, and amplification of the HER-2/neu oncogene. *Int. J. Cancer*, **47**, 66–71.

HARRIS, A. (1992). p53 Expression in human breast cancer. *Adv. Cancer Res.*, **59**, 69–88.

HURST, J., MANIAR, N., TOMBARKIEWICZ, J. & ROBERSON, C. (1991). Model of a human mammary tumour xenograft metastatic to lungs of nude mouse hosts. Presented at 14th Ann. San Antonio Breast Cancer Symp. Dec 5–7, 1991. *Breast Cancer Res. Treat.*, **19**, 184.

SCHIANO, M., SEVIN, B.U., PERRAS, J., RAMOS, R., WOLLOCH, E. & AVERETTE, H. (1991). *In vitro* enhancement of cis-platinium antitumour activity by caffeine and pentoxifylline in human ovarian cell line. *Gyn. Oncol.*, **43**, 37–45.